# Supplementary figures and images for: Functional heterogeneity in the fermentation capabilities of the healthy human gut microbiota
Source: PLoS One. 2021 Jul 21;16(7):e0254004. doi: 10.1371/journal.pone.0254004 (PMC8294568; doi:10.1371/journal.pone.0254004)

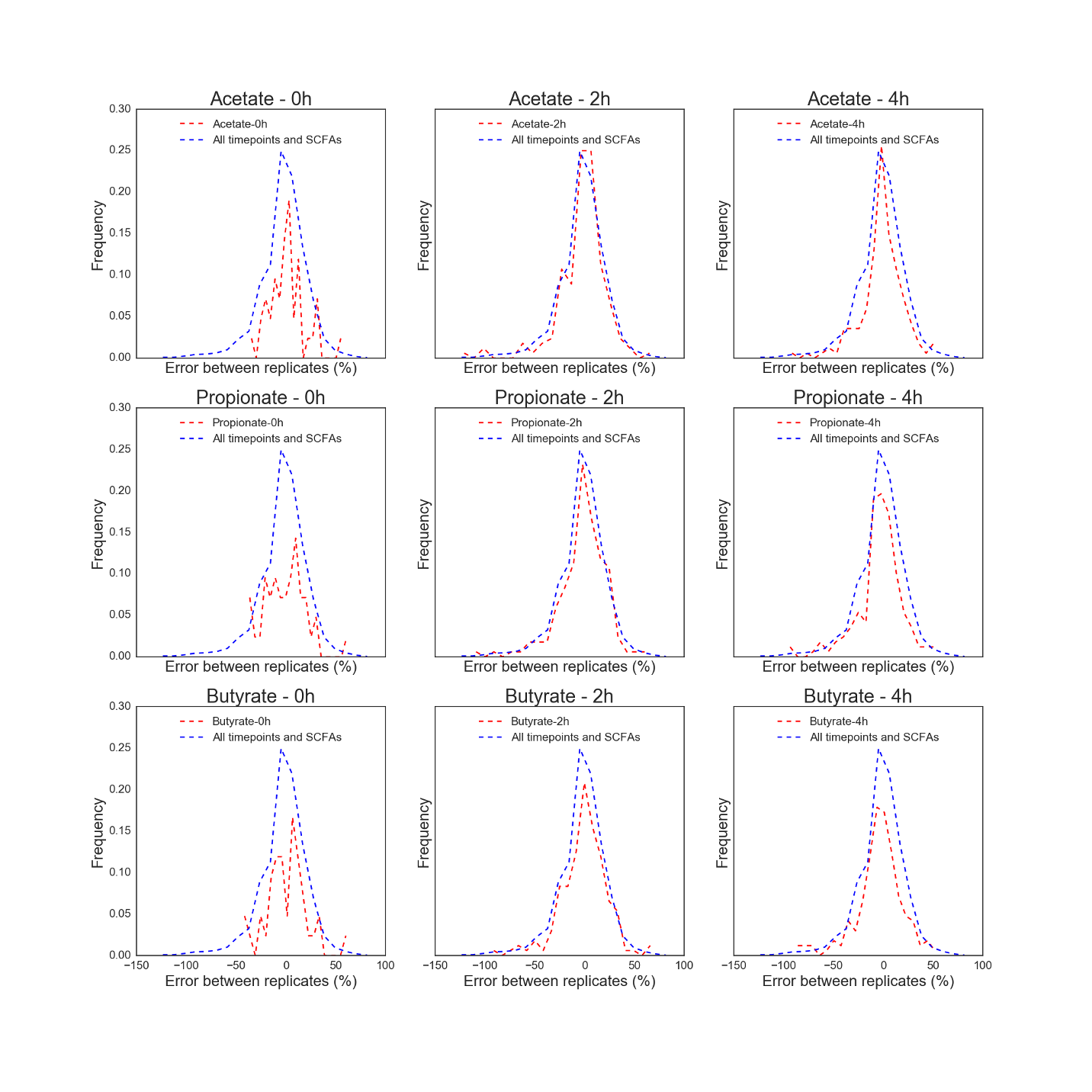

Supplement: S1 Fig — Percentage difference in SCFA quantification by GC-FID between biological replicates, broken down by timepoint and SCFA. The overall distribution, across all participants, timepoints, and SCFAs, is plotted in blue, while the individual distributions for each timepoint and SCFA are shown in red. (PNG) [file pone.0254004.s001.png]

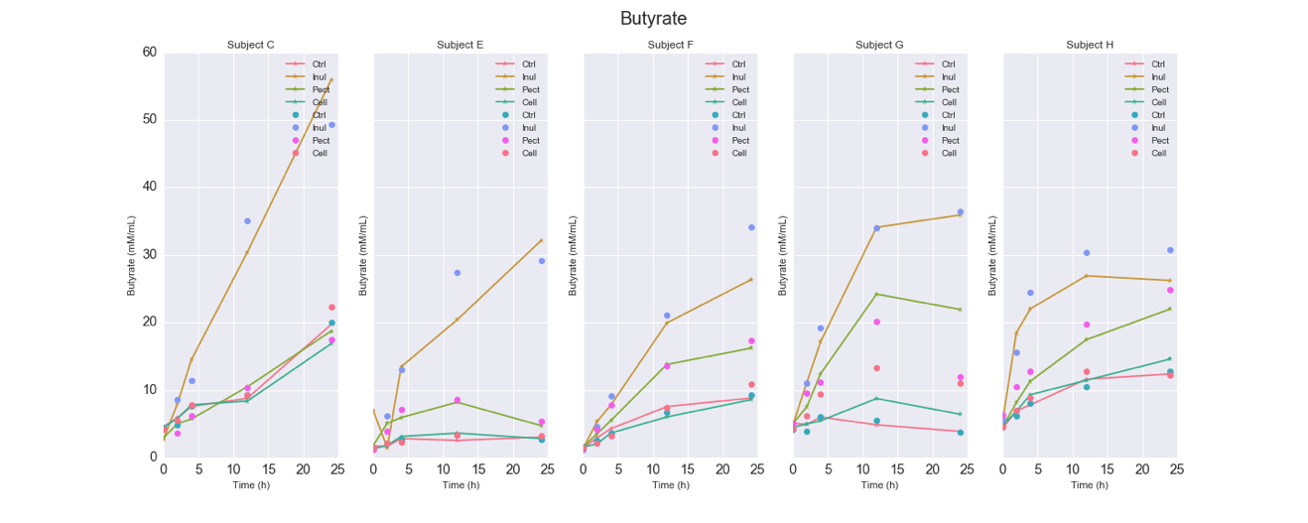

Supplement: S2 Fig — Individual 24h trajectories for Butyrate in five pilot participants. The different conditions are shown in different colors. (PNG) [file pone.0254004.s002.png]

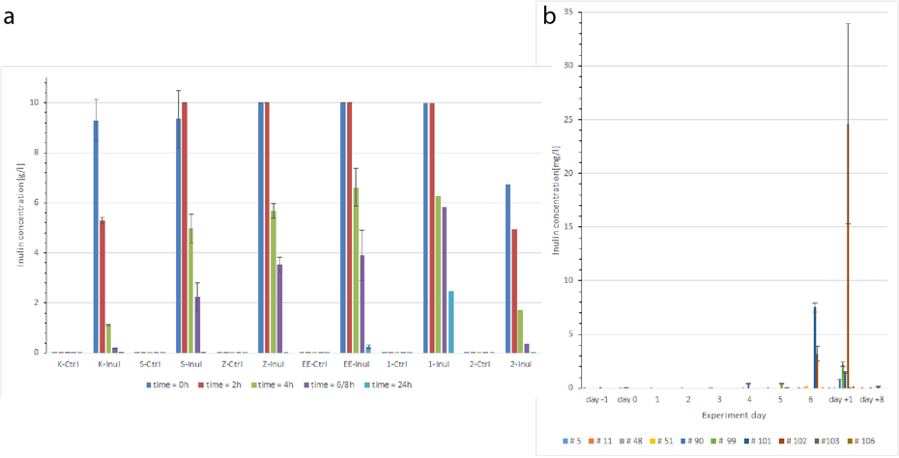

Supplement: S3 Fig — (a) Concentrations of inulin measured at t = 0, 2h, 4h, 6h and 24h in six separate donors (donor IDs K, S, Z, EE, 1 and 2), determined using an inulin-specific ELISA assay. (b) Concentration of inulin in participant stool from a previous study [21] where participants were fed 10g of inulin daily on days 4, 5 and 6 against a constant (fiber-impoverished) dietary background. Fecal inulin concentrations ranged from undetectable to a detectable level (maximum of 25 mg/L) on the days following inulin consumption. (PNG) [file pone.0254004.s003.png]

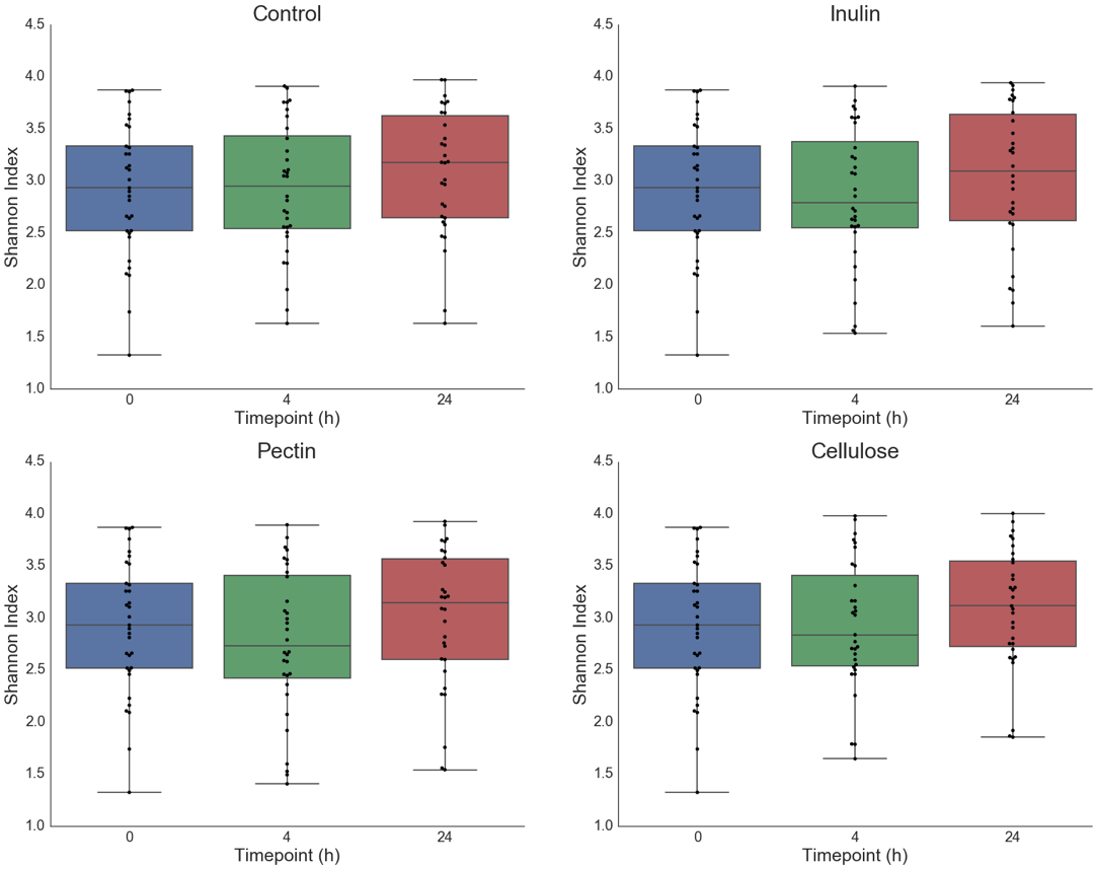

Supplement: S4 Fig — Shannon diversity index of 16S rRNA communities in each condition and each timepoint (0h, 4h, 24h). (PNG) [file pone.0254004.s004.png]

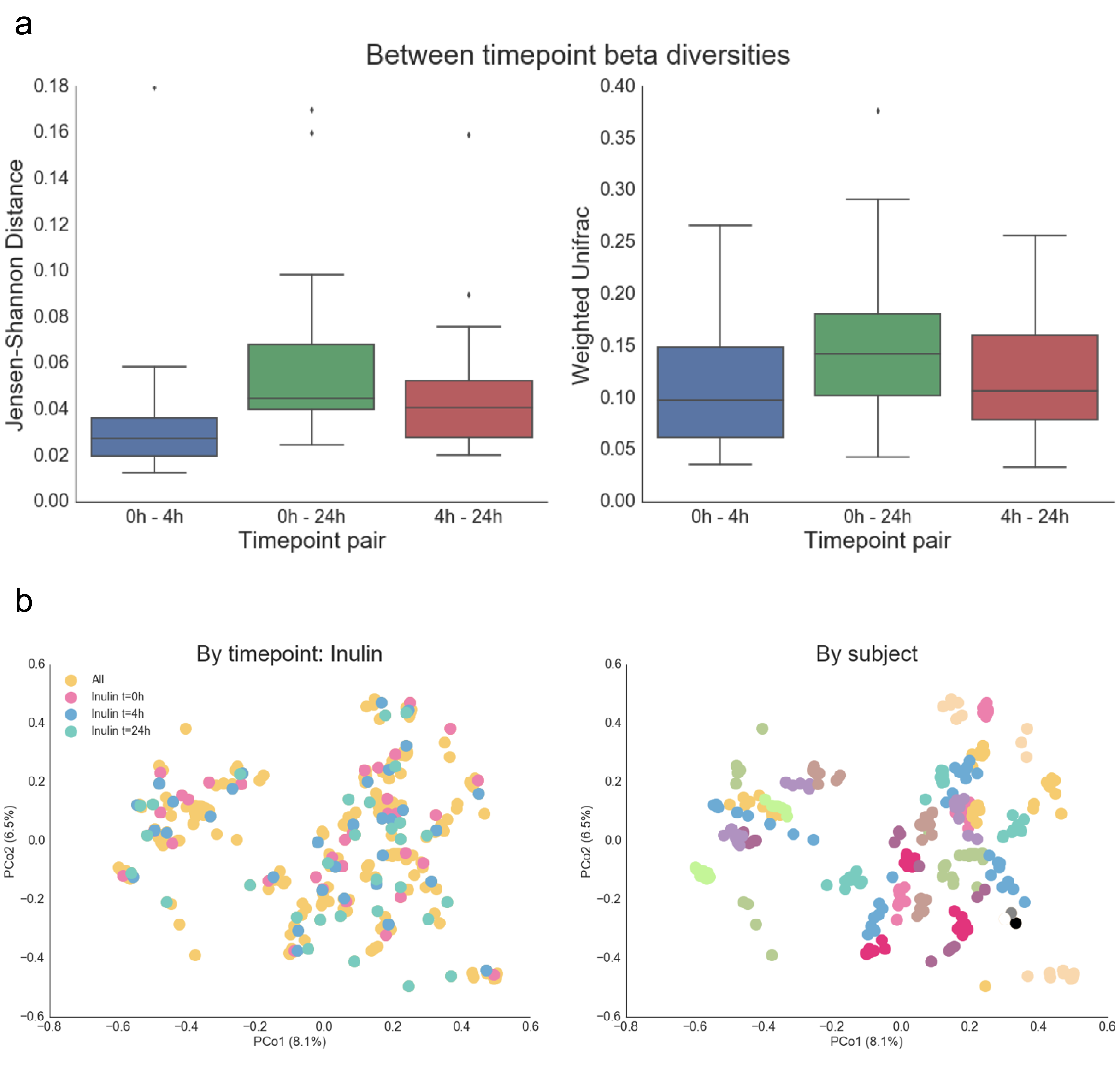

Supplement: S5 Fig — (a) Jensen-Shannon Distances (left) and Weighted Unifrac (right) beta-diversities between timepoints at the level of 16S rRNA (0-4h, 0-24h, 4-24h). (b) Multidimensional scaling analysis of all 16S rRNA samples, colored by timepoint (left) and by participant (right). (PNG) [file pone.0254004.s005.png]

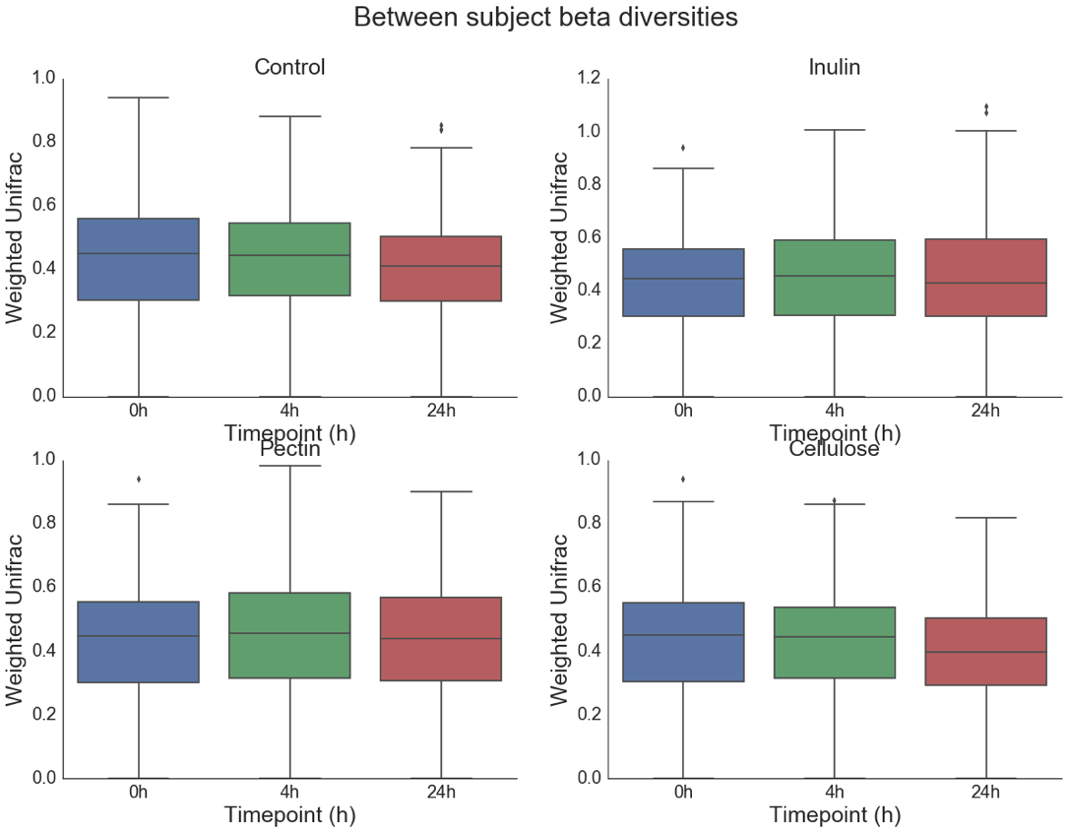

Supplement: S6 Fig — Between participant Weighted Unifracs at 0h, 4h and 24h. (PNG) [file pone.0254004.s006.png]

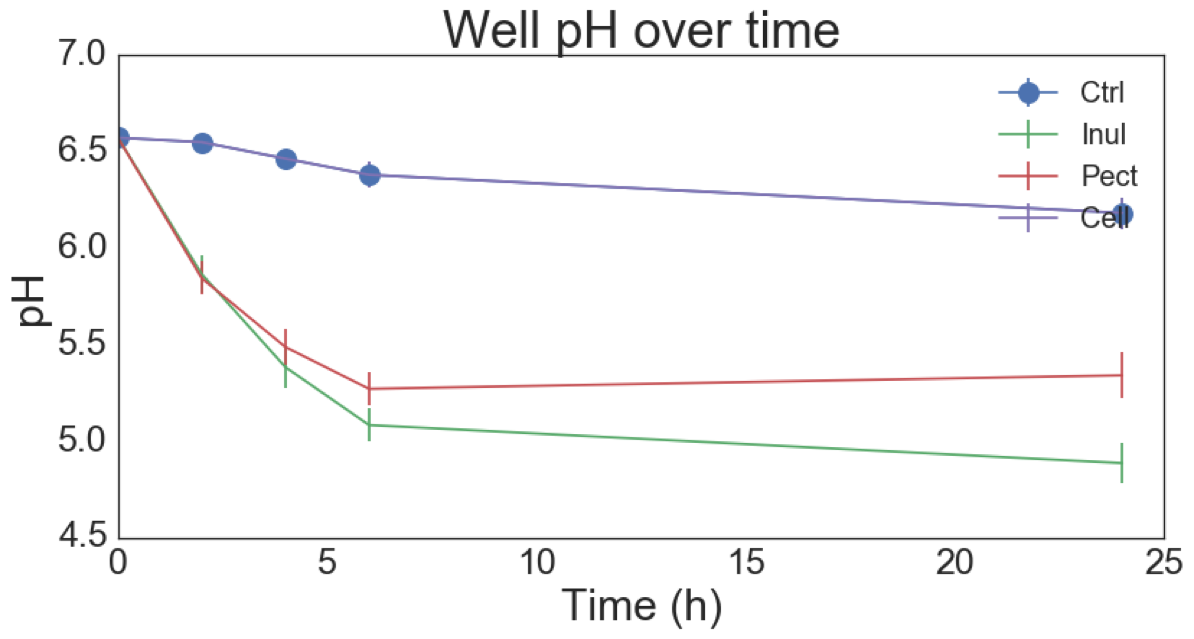

Supplement: S7 Fig — pH of the slurry over time, measured across all participants in the study. (PNG) [file pone.0254004.s007.png]

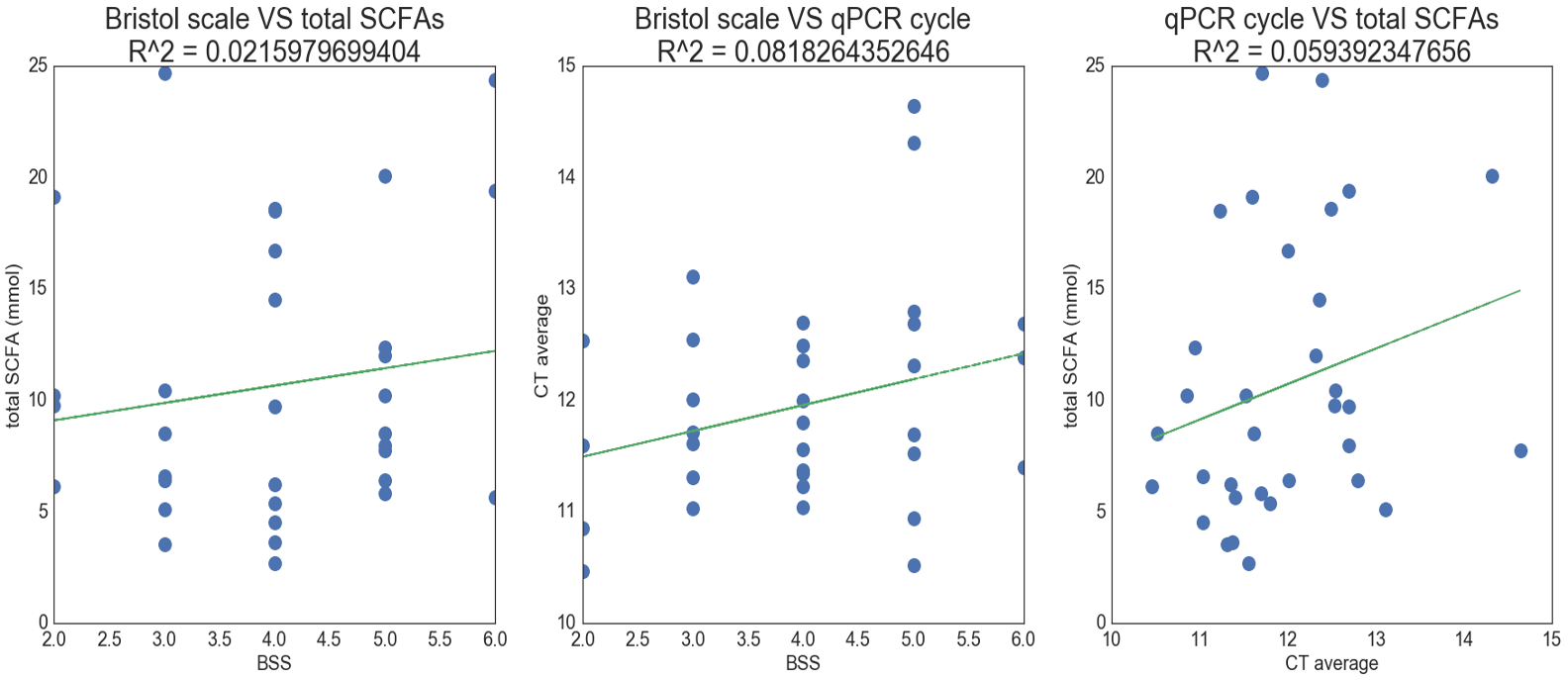

Supplement: S8 Fig — Pairwise linear regressions between between Bristol score of the sample, total SCFAs produced, and qPCR amplification cycle (CT values). (PNG) [file pone.0254004.s008.png]

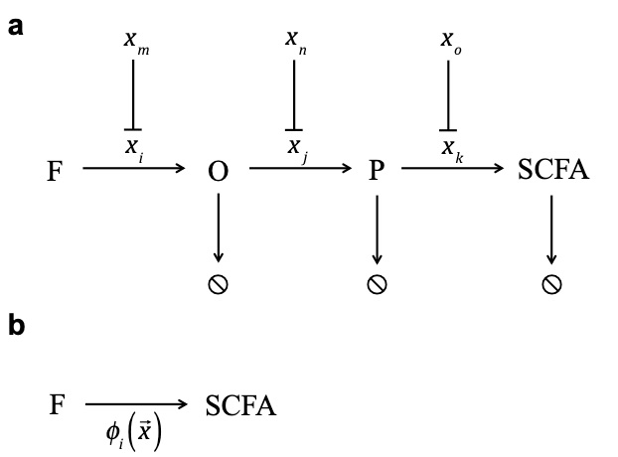

Supplement: S9 Fig — (a) Schematic illustrating the generic steps involved in dietary fiber degradation. A bacterial OTU i of relative abundance xi hydrolyses the polysaccharide (dietary fiber) F into oligosaccharides O. These are then fermented into a reduced intermediate P by OTU j with relative abundance xj. Finally, P may be further fermented to an SCFA by OTU k with relative abundance xk. In addition, the ability of an OTU to carry out a given reaction can itself be inhibited by a separate OTU (e.g. xi is inhibited by xo). (b) Bulk measurement of the overall production rate of a given SCFA, ϕSCFA(x), which is itself a function of the composition of the stool microbiota, x, and corresponds to the quantities measured using our ex vivo experiments. (PNG) [file pone.0254004.s009.png]

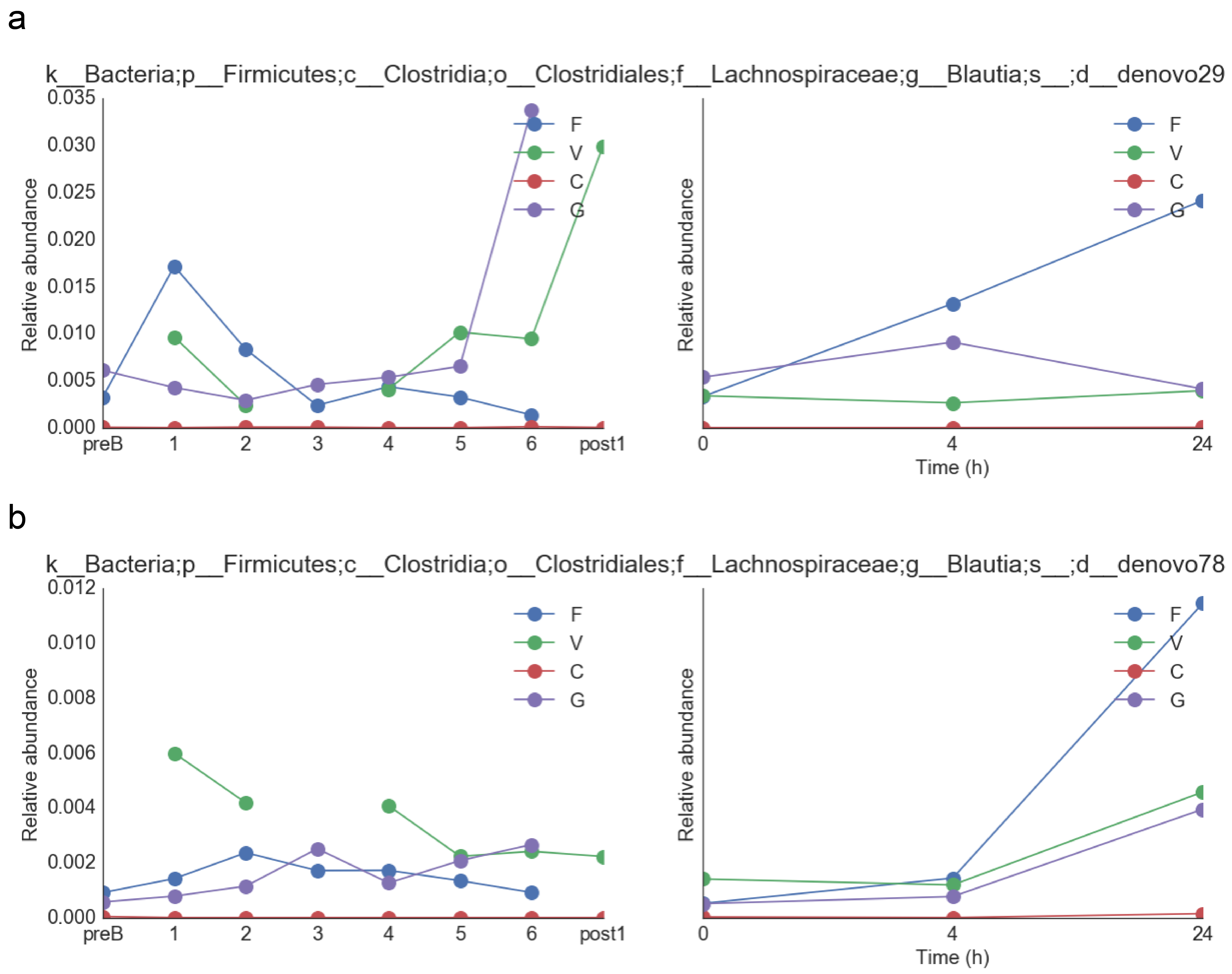

Supplement: S10 Fig — Comparison in relative abundances of two different OTUs of the genus Blautia in the same participants in vivo and ex vivo. (PNG) [file pone.0254004.s010.png]
