# Supplementary material for: Functional heterogeneity in the fermentation capabilities of the healthy human gut microbiota
Source: PLoS One. 2021 Jul 21;16(7):e0254004. doi: 10.1371/journal.pone.0254004 (PMC8294568; doi:10.1371/journal.pone.0254004)
Supplement: S1 File — (DOCX) [file pone.0254004.s012.docx]

**S1 File**

**Model of microbial SCFA production**

S9a Fig illustrates the steps in the degradation of dietary fibers into SCFAs that involve the microbiota. In this framework, distinct equations describing the time-evolution of the concentration of each chemical species exist for each type of monomer *M*, intermediate *P*, and SCFA. In reality, the system is sparse, as the rate constants *π_i1_*, *π_j2_*, and *π_k3_* are zero or close to zero in the vast majority of OTUs due to the fact that only a small subset of OTUs have the necessary biochemical capabilities. For a given concentration of dietary fiber [*F*], the combination of all contributing equations to a given rate of production of a specific SCFA can be summarized by a single parameter, , which is a function of the composition of the microbiota (S9b Fig). Thus, in *ex vivo* conditions,

.

**Estimation of absorbed SCFAs across the colon’s epithelium**

We approximate the colon as a cylinder 3cm in diameter. This corresponds to a circumference of approximately 9.42cm. Thus, each 1cm-thick cross-sectional segment of the colon has a surface area of approximately 9.42cm^2^, which we round up to 10cm^2^. Estimating the length of the colon at 150cm, we reach a total surface area of approximately 1,500cm^2^. Assuming the colon is filled with stool, we reach a total internal stool volume of 1,060cm^3^, which we approximate as 1L.

The absorption rate constant of a given SCFA estimated using the CaCo cell monolayer experiments, *ϒ_SCFA_*, is in units of mol cm^-2^ h^-1^L^-1^. Thus, the absorption rate of the entire colon is 1,500*ϒ_SCFA_* mol h^-1^L^-1^ of each SCFA. Based on our approximation of 1L of total stool, this amounts to an absorption rate of approximately 1,500*ϒ_SCFA_* mol h^-1^ for each SCFA.

**Comparison between *ex vivo* and *in vivo***

We next sought to determine whether changes in SCFAs or community structure observed under *ex vivo* conditions were in agreement with what could theoretically be measured *in vivo*. For this purpose, we turned to a dataset from a previous study in which participants were placed on a fixed diet consisting entirely of a fiber-impoverished, liquid, nutritional meal supplement for a period of six days [21]. In the latter three days, participants were randomized to a spike-in, to be consumed at a prescribed dose daily against the constant liquid diet background. These spike-ins included inulin, pectin, and cellulose, and used the exact same sources of these three fibers, providing us with an ideal comparison dataset.

Under *ex vivo* conditions, we observed complete degradation of the inulin bolus in certain participants (Fig 3a). We sought to determine whether a similar extent of degradation could be observed *in vivo* in participants consuming an inulin bolus at similar concentrations. Residual inulin was therefore quantified from the stool using an inulin-specific ELISA assay. We find that, as expected, no detectable inulin can be found in the stool of participants on days 1, 2 and 3, but that inulin is detectable on days after which inulin was consumed in certain stool samples (Fig 4b). However, the inulin detected on these days accounts for a tiny minority of the total inulin consumed (10g/day), which assuming a total daily stool volume of 1L, equates to approximately 10g/L, the concentration used in the *ex vivo* experiments. This suggests that the majority of accessible inulin is also consumed *in vivo* in the typical stool passage time following ingestion. Moreover, since 10g is a significantly larger dose of inulin than would ordinarily be consumed in a typical diet, we can conclude that the majority of inulin consumed in an ordinary diet in a form similarly accessible to the inulin powder used for these experiments is fermented *in vivo*. Of course, it is likely that inulin from natural sources and typical dietary fiber sources do not contain the fiber in as accessible form as the purified form used in these experiments.

In order to assess the extent to which the *ex vivo* conditions result in the growth of different organisms compared to *in vivo*, we recruited the same participants from the *in vivo* study that had consumed inulin as a spike-in, performed an *ex vivo* experiment, and directly compared the 16S rRNA data between the two so the comparison could be made within the same person. More specifically, we compared OTUs changed *in vivo* from day 3 to day 6, or changed *ex vivo* from *t*=0 to *t*=24h, and plotted their trajectories next to each other (S10 Fig). Comparing two separate OTUs of the same butyrate-producing genus, *Blautia*, we find that participant OTU denovo29 blooms in participants G and V *in vivo* but in participant F *ex vivo*, while OTU denovo78 blooms in all participants *ex vivo* but does not change appreciably *in vivo*. Thus, these data argue that *ex vivo* conditions may introduce artefacts in growth rates of different bacteria compared to the gut, further arguing for measurement in the first hours of the experiment while the stool community remains relatively unchanged.
